# Estimation of Severe Middle East Respiratory Syndrome Cases in the Middle East, 2012–2016

**DOI:** 10.3201/eid2210.151121

**Published:** 2016-10

**Authors:** Justin J. O’Hagan, Cristina Carias, Jessica M. Rudd, Huong T. Pham, Yonat Haber, Nicki Pesik, Martin S. Cetron, Manoj Gambhir, Susan I. Gerber, David L. Swerdlow

**Affiliations:** IHRC Inc., Atlanta, Georgia, USA (J.J. O’Hagan. C. Carias);; Centers for Disease Control and Prevention, Atlanta (J.J. O’Hagan, C. Carias, J.M. Rudd, H.T. Pham, Y. Haber, N. Pesik, M.S. Cetron, M. Gambhir, S.I. Gerber, D.L. Swerdlow);; Imperial College London, London, UK (M. Gambhir);; Monash University, Melbourne, Victoria, Australia (M. Gambhir)

**Keywords:** Middle East respiratory syndrome, MERS-CoV, coronavirus, viruses, incidence, travel, population surveillance, disease outbreaks, zoonoses, South Korea, respiratory infections, Middle East

## Abstract

Using data from travelers to 4 countries in the Middle East, we estimated 3,250 (95% CI 1,300–6,600) severe cases of Middle East respiratory syndrome occurred in this region during September 2012–January 2016. This number is 2.3-fold higher than the number of laboratory-confirmed cases recorded in these countries.

## Abstract 2

Our traveler-based estimate was 2.3-fold higher than the number of laboratory-confirmed cases recorded.

Middle East respiratory syndrome (MERS), caused by MERS coronavirus (MERS-CoV), was first recognized in September 2012 (*1*). From that time until January 2016, >1,600 cases were laboratory-confirmed, and ≈600 deaths have been attributed to the virus (*2*). Cases have been detected among persons who traveled from the Middle East to 16 countries, and a MERS-CoV outbreak in South Korea introduced by a traveler caused >100 cases (*3*).

Estimates of the epidemic size in the Middle East are required to understand the level of MERS-CoV circulation and the likelihood of MERS-CoV exportations. However, these estimates have not been calculated for >2 years, during which time the number of recorded cases has increased by >15 times (*2*,*4*). We used data from travelers to this region to update estimates of severe MERS cases in the Middle East.

## The Study

We estimated the cumulative number of severe MERS cases in source countries from which laboratory-confirmed MERS-CoV infections were reported by the World Health Organization for nonresident travelers during September 2012–January 2016. The source countries were Saudi Arabia, United Arab Emirates, Jordan, and Qatar (*1*); only the emirates of Abu Dhabi and Dubai were included in the United Arab Emirates calculations due to a lack of traveler data on the other 5 emirates, but no MERS cases have been reported from these other emirates.

All MERS traveler case-patients whose data were used in the analysis were hospitalized because of respiratory symptoms and therefore likely experienced severe disease. However, 30% of reported MERS case-patients were listed as mildly symptomatic or asymptomatic and were likely missed by the passive surveillance systems that detected severe travel-associated cases (*1*). Consequently, we used the term “severe” to indicate that our estimates of case numbers are for those with more serious disease.

To estimate the cumulative number of severe cases, we used data for 1) the number of travelers to the 4 source countries, 2) average trip lengths, 3) number of confirmed MERS-CoV infections among travelers to the source countries, and 4) population sizes of source countries. Our estimates are for the period September 2012, when MERS-CoV was first identified, through January 2016.

We estimated the number of severe cases in each source country using methods used previously by Cauchemez et al., which assume that travelers and local residents have similar per-day risk of infection (*4*). The infection rate among travelers to source countries was multiplied by the total person-time at risk for the population of that country using the following formula:

cumulative number of severe MERS-CoV cases in country X = 

severe case rate among travelers to source countries × country X person-time =

×country X population size × 365 × epidemic periodwhere the epidemic period = 3.33 years (September 2012–January 2016). We estimated CIs using profile-likelihoods (*4*). This approach can estimate the cumulative incidence of disease regardless of seasonality in infection rates (*5*). 

We analyzed 11 travel-associated MERS cases, including 6 case-patients from high-income countries (Technical Appendix). It has been suggested that MERS-CoV surveillance may be better in high-income countries than in lower-income countries (*6*). We tested this hypothesis by comparing the frequency of case detection among travelers returning to high-income countries, as defined by the Organization for Economic Cooperation and Development (http://www/oecd.org/about/membershipandpartners/), versus lower-income countries (all other non–Middle Eastern countries worldwide). We found that significantly more cases have been identified in high-income settings (p<0.001 by Fisher exact test; Technical Appendix). Consequently, we produced 2 sets of calculations, 1 using data only from high-income countries and 1 that combined data from all non–Middle Eastern countries. The high-income country analysis does not assume that no cases occurred in lower-income countries but shows different case detection rates across travelers’ home countries.

Using data for 32 high-income countries, we estimated ≈3,263 severe cases (95% CI 1,297–6,613; Table 1) for all source countries during September 2012–January 2016. We calculated that Saudi Arabia had the largest number of cases (2,269, 95% CI 902–4,599). We estimated ≈1,431 severe cases (95% CI 743–2,452) when data from high-income and lower-income countries were combined.

**Table 1:** Estimated cumulative incidence of severe Middle East respiratory syndrome cases in Middle Eastern source countries calculated on the basis of illnesses among travelers, September 2012–January 2016*

Traveler origins	Estimated no. cases (95% CI)				
	All countries	Saudi Arabia	Jordan	Qatar	UAE†
Visitors from high-income OECD countries	3,263 (1,297–6,613)	2,269 (902–4,599)	483 (192–979)	163 (65–330)	347 (138–704)
All non–Middle Eastern visitors	1,431 (743–2,452)	995 (517–1,705)	212 (110–363)	72 (37–123)	152 (79–261)

*OECD, Organization for Economic Cooperation and Development; UAE, United Arab Emirates. †Only the emirates of Abu Dhabi and Dubai were included in calculations due to a lack of traveler data on the other 5 emirates; no cases have been reported from these other emirates.

We conducted sensitivity analyses in which we included 1) laboratory-confirmed cases among travelers for whom it was unclear in which country they had been infected or if they had been infected by another travel-associated case-patient and 2) probable but non–laboratory-confirmed MERS-CoV cases reported in travelers. These analyses indicated there could have been up to 4,895 severe cases across source countries (95% CI 2,352–8,824). We also conducted sensitivity analyses to assess the effect of uncertainty of travelers’ average length of stay in source countries (Table 2; Technical Appendix Tables 2, 3). Increases in travelers’ assumed lengths of stay produced lower cumulative incidence estimates related to lower estimated infection rates, and decreases in travelers’ assumed lengths of stay produced higher case estimates. For example, using data for travelers from high-income nations, a 2-day increase in average length of stay produced estimates of 2,326 severe cases across source countries (95% CI 924–4,714; Technical Appendix Table 2), and a 2-day decrease in lengths of stay produced estimates of 5,463 severe cases (95% CI 2,171–11,071).

**Table 2:** Estimated cumulative incidence of severe Middle East respiratory syndrome cases in Middle Eastern source countries calculated on the basis of illnesses among travelers and traveler LOS, September 2012–January 2016*

	Estimated no. cases (95% CI)				
Traveler data	–2 Days LOS	–1 Day LOS	Average LOS†	+1 Day LOS	+2 Days LOS
Visitors from high-income OECD countries	5,463 (2,171–11,071)	4,086 (1,623–8,280)	3,263 (1,297–6,613)	2,716 (1,079–5,504)	2,326 (924–4,714)
All non–Middle Eastern visitors	2,043 (1,061–3,499)	1,683 (874–2,883)	1,431 (743–2,452)	1,245 (647–2,132)	1,102 (572 – 1,887)

*LOS, length of stay; OECD, Organization for Economic Cooperation and Development. †The average length of stay of travelers from OECD countries in the 4 source countries (Saudi Arabia, Jordan, Qatar, and United Arab Emirates) was estimated to be 5.0 d, and the average length of stay of travelers from all non–Middle Eastern countries in the 4 source countries was estimated to be 6.7 d (Technical Appendix). Only the emirates of Abu Dhabi and Dubai were included in United Arab Emirates calculations due to a lack of traveler data on the other 5 emirates; no cases have been reported from these other emirates.

## Conclusions

We used data on the incidence of MERS among travelers returning from the Middle East to better estimate the occurrence of severe disease in the most affected countries. We estimated that there were ≈3,300 cases of severe disease in the 4 source countries during September 2012–January 2016. This estimate was 2.3-fold higher than the total number of laboratory-confirmed cases across source countries from September 2012–January 2016. 

Using data up to August 2013, Cauchemez et al. estimated the total case count to be 11-fold higher than the number of laboratory-confirmed cases reported across source countries (*4*). The closer agreement between observed and estimated cases in our analysis is consistent with improvements in surveillance practices across source countries during 2014 (*6**–**8*). Our results are also complementary to a serologic study from Saudi Arabia that reported antibodies to MERS-CoV were found in 0.15% of the population (*10*). Our study adds information by focusing on severe infections (which are of greatest clinical concern) and providing more up-to-date information by including data from the 2-year period after the serologic samples were collected.

Our estimates were based on a small sample size (11 travel-associated cases) and assumed that travelers and residents of the Middle East had similar infection risks. Our sensitivity analyses demonstrated that results are sensitive to travelers’ estimated lengths of stay and also showed that estimates of the epidemic size that incorporated data from lower-income countries were 60% lower than estimates obtained by using data from high-income countries alone. This finding implies different levels of case detection across travelers’ home countries or different MERS-CoV exposure between visitors of different nationalities. Additional data (e.g., larger sample size, travel volume, and lengths of stay, stratified by age and immigration status, frequencies of testing, and contact with camels) could provide further estimates.

Public health officials are concerned about MERS-CoV, both in the source countries and from exported cases in persons who can seed outbreaks elsewhere (*9*,*11*). By better estimating the epidemic size in the Middle East, our results can help guide public health preparedness efforts in source countries and contribute to projections of the number of cases that could occur among travelers (*9*,*11*–*13*).

Technical Appendix Additional information and statistical analysis for estimation of severe Middle East respiratory syndrome cases in the Middle East, 2012–2016.
