# Supplementary material for: Estimation of Severe Middle East Respiratory Syndrome Cases in the Middle East, 2012–2016
Source: Emerg Infect Dis. 2016 Oct;22(10):1797–9. doi: 10.3201/eid2210.151121 (PMC5038414; doi:10.3201/eid2210.151121)
Supplement: Technical Appendix — Additional information and statistical analysis for estimation of severe Middle East respiratory syndrome cases in the Middle East, 2012–2016. [file 15-1121-Techapp-s1.pdf]

Article DOI: http://dx.doi.org/10.3201/eid2210.151121

# Estimation of Severe MERS-CoV Cases in the Middle East, 2012–2016

## Technical Appendix

## Data

To estimate the cumulative number of severe cases in source countries (i.e., Kingdom of Saudi Arabia [KSA], United Arab Emirates [UAE], Jordan, and Qatar), we used data on 1) number of travelers to the 4 source countries, 2) travelers' average trip lengths, 3) number of laboratory-confirmed MERS-CoV cases among travelers to source countries, and 4) source countries' population sizes.

Data on traveler numbers were obtained from World Trade Organization (WTO) publications and UAE and Qatar visitor information (*1*–*5*, Technical Appendix Table 1). We estimated the number of persons traveling to the source countries from high-income countries, where "high-income" was defined as those countries belonging to the Organization for Economic Cooperation and Development (OECD) and which were not listed as "emerging economies" (i.e., Mexico, Chile, and Turkey were classified as lower-income countries along with all non-OECD countries) (*6*). These high-income OECD countries could have different degrees of MERS-CoV case detection than other states since the former have more resources for identifying the etiology of illnesses among returning travelers (*7*,*8*). We used WTO data on traveler numbers for Jordan and KSA, which collated information on the number of travelers arriving at their borders (*2*). UAE traveler numbers were obtained from official counts of hotel visitors to Dubai (2012 data) and Abu Dhabi (first quarters of 2013 and 2014, which we averaged and scaled to annual data) (*3*,*4*). Information for Qatar was obtained from government data on the number of visas granted to travelers from different regions (*5*). Cases in UAE have only been reported from Abu Dhabi and Dubai and they are the most populous emirates in UAE (*9*,*10*). Therefore, we excluded the other 5 UAE emirates from consideration since data on their traveler numbers were not available and they have not reported cases up to January 2016. We assumed that the number of travelers is similar over the epidemic period as it was in 2012 because data were not yet available for 2013–14.

Travelers' average trip lengths were obtained from a WTO publication (Jordan and Qatar, 2012 data), hotel stay data (Abu Dhabi 2014 data, which we also applied to Dubai visitor numbers), and KSA tourism data from 2011 (*1*,*4*,*11*). Many visitors spend more than a month in KSA, which we accounted for in our calculations since such visitors could become infected and recover by the time they travel home. We used 14 days as the upper limit of the period before traveling home during which visitors were at risk for contracting a MERS-CoV infection that could be detected upon arrival in their home country. We chose 14 days since the average incubation period has been

estimated as 5.5 days and travel-associated cases were tested for MERS-CoV an average of 9 days after symptom onset (*12*). For durations of stay less than 14 days, we used the midpoint of the trip length categories in calculations (*11*). The overall average duration of stay for travelers to KSA was then estimated by summing the durations of stay of all travelers from non-Middle Eastern countries and dividing by the total number of these travelers. Separate estimates of the average duration of stay were calculated for travelers from high-income OECD countries where possible.

The number of laboratory-confirmed MERS-CoV cases among travelers to source countries was obtained from publicly available information (*13*). When estimating the number of severe cases in source countries, we excluded cases among travelers that occurred among foreign residents or citizens of a source country or that were detected as a result of testing before departure from a source country (*14*). We excluded these cases since foreign residents and citizens of source countries likely have different travel patterns to visitors (e.g., they may postpone travel if ill), their length of stay in their destination could be shorter than the incubation period, they may wait to seek care until they return home, and data on their travel frequency were also unavailable. MERS-CoV cases who were transported to another country for medical care were also excluded because they occurred among citizens of source countries and they are not representative of average infection risk since their travel was related to their infection status.

Finally, countries' population sizes were obtained from national estimates (*9,10,15–17*) (Technical Appendix Table 1).

## Analyses

The cumulative number of severe MERS-CoV cases was estimated by using methods described previously (*12*). First, the rate of severe MERS-CoV disease among travelers was calculated by dividing the number of detected cases among this group by their total time at risk in the source countries. Second, the cumulative number of severe cases in each source country was then estimated by multiplying the infection rate among travelers by the total person-time among the resident population according to the following formula:

Cumulative number of severe cases in source country X =

Severe case rate among travelers to all source countries × Person-time of country X =

$$\frac{\text{number of severe cases reported among travelers returning from source countries}}{\text{annual number of travelers to source countries} * \text{average trip length} \times \text{epidemic period}} \times$$

Source country X population size × 365 × epidemic period =

$$\frac{\text{number of severe cases reported among travelers returning from source countries}}{\text{annual number of travelers to source countries} * \text{average trip length}} \times$$

Source country X population size × 365

Here, the "epidemic period" corresponds to 3.33 years (September 2012 – January 2016). Notably, the length of the epidemic period cancels in the equation and so did not affect calculations. Our estimates of the cumulative number of severe MERS-CoV cases are for the period September 2012, when MERS-CoV was first identified, through January 2016. As described elsewhere, the average rate used by the formula can give appropriate estimates of the cumulative incidence of disease even if the rate of infection varies over time as long as the person-time at risk is uniformly distributed over the outbreak (*18*). The calculation assumes that the disease rate is the same across source countries. Analyses using country-specific disease rates could be performed with larger numbers of travel-associated cases. 95% confidence intervals were calculated by using profile-likelihoods (*19*). Matlab 2014a was used for statistical analyses.

To determine whether levels of surveillance for MERS-CoV differed between travelers' home countries, we compared the frequency with which MERS-CoV cases have been identified in high-income OECD countries versus all other non-Middle Eastern countries. We only included cases that were identified by using passive surveillance since these better reflect surveillance capacity than cases that were identified because authorities were alerted to the arrival of a possibly MERS-CoV infected person by officials in a source country. Infections that occurred among residents or citizens of source countries which were identified in non-Middle Eastern countries were included in this analysis since the goal was simply to compare rates of passive surveillance between groups of non-Middle Eastern countries. There were 10 such MERS-CoV importation events identified in high-income countries compared to 8 importation events detected in lower-income countries from September 2012 – January 2016 (Technical Appendix Table 1). The total number of air travelers to each group of destination countries from source countries was obtained from published estimates (8). There was a significant difference in the frequency of detection of cases between groups of countries ($p < 0.001$ by Fisher exact test).

## Sensitivity analyses

It was unclear whether travel-associated cases with unconfirmed infection or who were possibly infected by another traveler should be included. For example, a second Dutch case was found after a contact investigation of an index case (*20*), but it was unclear where the second case's infection occurred and whether they acquired it from the index case. Consequently, the second case might not provide independent information on the size of the MERS-CoV epidemic in KSA. Additionally, 2 probable, but unconfirmed, MERS-CoV cases occurred in late 2013 among travelers returning from KSA to Spain (*21*), and both belonged to the same tour group making it unclear whether they were potentially separate infections or if one could have infected the other. These persons were included in sensitivity analyses for all high-income countries as well as for estimates calculated by using data from all non-Middle Eastern countries.

We conducted an additional sensitivity analysis to highlight how the results depend on estimates of travelers' average lengths of stay in the source countries. To do so, we recalculated the expected number of severe MERS-CoV cases across source countries using a range of average durations of stay of travelers from OECD

countries and all non-Middle Eastern countries from 2 days below the point estimates obtained from countries' visitor data to 2 days above the point estimates (Technical Appendix Tables 2–3).

## References

1. World Tourism Organization. Compendium of Tourism Statistics, Data 2008-2012, 2014 Edition. Madrid (Spain). http://www.e-unwto.org/doi/book/10.18111/9789284415939

2. World Tourism Organization. Yearbook of Tourism Statistics, Data 2008–2012, 2014 edition. Madrid (Spain) http://www.e-unwto.org/doi/book/10.18111/9789284415915

3. Dubai Department of Tourism & Commerce Marketing. Dubai Hotel Statistics 2012 [cited 2014 Jun 4]. http://www.dubaitourism.ae/trade-resources/statistics/hotel-statistics

4. Abu Dhabi Tourism and Culture Authority. Hotel Guest Data [cited 2014 Jun 4]. http://tcaabudhabi.ae/en/about/Pages/reports-and-statistics.aspx

5. Qatar Tourism Authority - Midyear Report (Jan - Jun) (2013–2014). 2014 [cited 2015 Jun 19]. http://www.justhere.qa/2014/10/press-release-qatar-tourism-industry-surged-ahead-first-half-2014/

6. Organization for Economic Cooperation and Development. Member countries [cited2014 Jun 10]. http://www.oecd.org/about/membersandpartners/

7. Khan K, McNabb SJ, Memish ZA, Eckhardt R, Hu W, Kossowsky D, et al. Infectious disease surveillance and modelling across geographic frontiers and scientific specialties. Lancet Infect Dis. 2012;12:222–30. http://dx.doi.org/10.1016/S1473-3099(11)70313-9

8. Khan K, Sears J, Hu VW, Brownstein JS, Hay S, Kossowsky D, et al. Potential for the international spread of Middle East respiratory syndrome in association with mass gatherings in Saudi Arabia. PLoS Curr. 2013:5. http://dx.doi.org/10.1371/currents.outbreaks.a7b70897ac2fa4f79b59f90d24c860b8

9. Government of Dubai Statistics Center. Population size [cited 2014 Jun 4]. https://www.dsc.gov.ae/en-us/Publications/Pages/publication-details.aspx?PublicationId=5

10. Statistics Centre – Abu Dhabi. Population size (mid-2012 estimate) [cited 2014 Jun 4]. https://www.scad.ae/en/Pages/ThemesReleases.aspx?ThemeID=4

11. Saudi Commission for Tourism & Antiquities. Tourism Statistics 2011 [in Arabic] [cited 2014 May 30]. http://www.mas.gov.sa/

12. Cauchemez S, Fraser C, Van Kerkhove MD, Donnelly CA, Riley S, Rambaut A, et al. Middle East respiratory syndrome coronavirus: quantification of the extent of the epidemic, surveillance biases, and transmissibility. Lancet Infect Dis. 2014;14:50–6. http://dx.doi.org/10.1016/S1473-3099(13)70304-9

13. World Health Organization. Emergencies, preparedness, response. Coronavirus Infections Disease Outbreak News [cited 2015 Jun 30]. http://www.who.int/csr/don/archive/disease/coronavirus_infections/en/

14. World Health Organization. Middle East respiratory syndrome coronavirus (MERS-CoV) – update, April 17 2014. [cited 2014 Jun 8]; http://www.who.int/csr/don/2014_04_17_mers/en/

15. Kingdom of Saudi Arabia Central Department of Statistics and Information. Total population 2013 [in Arabic]. [cited 2014 Jun 3]; http://www.cdsi.gov.sa/english/index.php

16. Jordanian Department of Statistics. Population estimate (2012) [in Arabic](6). [cited June 3 2014]; http://www.dos.gov.jo/dos_home_a/main/yearbook_2012.pdf

17. Qatar Ministry of Development Planning and Statistics. Population Estimate (31 May 2014) [in Arabic] [cited June 3 2014]. http://www.qsa.gov.qa/eng/PopulationStructure.htm

18. Fraser C, Donnelly CA, Cauchemez S, Hanage WP, Van Kerkhove MD, Hollingsworth TD, et al. Pandemic potential of a strain of influenza A (H1N1): early findings. Science. 2009;324:1557–61. http://dx.doi.org/10.1126/science.1176062

19. Cole SR, Chu H, Greenland S. Maximum likelihood, profile likelihood, and penalized likelihood: a primer. Am J Epidemiol. 2014;179:252–60. http://dx.doi.org/10.1093/aje/kwt245

20. Kraaij-Dirkzwager M, Timen A, Dirksen K, Gelinck L, Leyten E, Groeneveld P, et al. Middle East respiratory syndrome coronavirus (MERS-CoV) infections in two returning travellers in the Netherlands, May 2014. Euro Surveill. 2014;19(21). http://www.eurosurveillance.org/ViewArticle.aspx?ArticleId=20817 **PMID: 24906375**

21. World Health Organization. Middle East respiratory syndrome coronavirus (MERS-CoV) summary and literature update–as of 20 January 2014 [cited 2014 Aug 6]. http://www.who.int/csr/disease/coronavirus_infections/MERS_CoV_Update_20_Jan_2014.pdf?ua=1

22. World Health Organization. Coronavirus infections; Disease outbreak news 24 May 2015 [cited 2015 Jun 23]. http://www.who.int/csr/don/archive/disease/coronavirus_infections/en/

23. World Health Organization. Middle East respiratory syndrome coronavirus (MERS-CoV) – Republic of Korea, May 24 2015 [cited 2015 Jun 16]. http://www.who.int/csr/don/24-may-2015-mers-korea/en/

24. World Health Organization. Middle East respiratory syndrome coronavirus (MERS-CoV) – Austria; Disease outbreak news 2 October 2014 [cited 2015 Jun 23]. http://www.who.int/csr/don/02-october-2014-mers-austria/en/

25. Tsiodras S, Baka A, Mentis A, Iliopoulos D, Dedoukou X, Papamavrou G, et al. A case of imported Middle East respiratory syndrome coronavirus infection and public health response, Greece, April 2014. Euro Surveill. 2014;19(16):20782. http://dx.doi.org/10.2807/1560-7917.ES2014.19.16.20782

26. Bialek SR, Allen D, Alvarado-Ramy F, Arthur R, Balajee A, Bell D, et al. First confirmed cases of Middle East respiratory syndrome coronavirus (MERS-CoV) infection in the United States, updated information on the epidemiology of MERS-CoV infection, and guidance for the public, clinicians, and public health authorities - May 2014. MMWR Morb Mortal Wkly Rep. 2014;63:431–6.

27. World Health Organization. Middle East respiratory syndrome coronavirus (MERS-CoV) – Turkey, October 24 2014 [cited 2015 Jun 18]. http://www.who.int/csr/don/24-october-2014-mers/en/

28. World Health Organization. Middle East respiratory syndrome coronavirus (MERS-CoV) – Thailand, January 29 2016. [cited Feb 2 2016]. http://www.who.int/csr/don/29-january-2016-mers-thailand/en/

29. World Health Organization. Middle East respiratory syndrome coronavirus (MERS-CoV) – Thailand, June 20 2015. [cited Feb 2 2016]. http://www.who.int/csr/don/20-june-2015-mers-thailand/en/

30. Drosten C, Seilmaier M, Corman VM, Hartmann W, Scheible G, Sack S, et al. Clinical features and virological analysis of a case of Middle East respiratory syndrome coronavirus infection. Lancet Infect Dis. 2013;13:745–51. http://dx.doi.org/10.1016/S1473-3099(13)70154-3

**Technical Appendix Table 1.** Population sizes, numbers of inbound travelers, and number of MERS-CoV cases among travelers for United Arab Emirates*, Jordan, Qatar, and Kingdom of Saudi Arabia†‡

| Country | Population size | Annual no. of inbound travelers from non-Middle Eastern countries | Annual no. of inbound travelers from high-income OECD countries§¶ # | No. exported cases**†† | |
|---|---|---|---|---|---|
| Abu Dhabi and Dubai | 4,592,777 | 7,934,133 | 3,922,345 | 2 (France, Germany – included in main analysis) | |
| Jordan | 6,388,000 | 2,155,000 | 864,944 | 1 (Italy – included in main analysis) | |
| Qatar | 2,155,446 | 1,807,638 | 594,902 | 1 (Tunisia – included in main analysis) | |
| Kingdom of Saudi Arabia | 29,994,272 | 6,213,000 | 446,267 | Included in main analysis | 6 (United Kingdom, Malaysia, Netherlands, Algeria x 2, The Philippines) |
| | | | | Included in sensitivity analyses | 3 (Netherlands, Spain x 2) |

*Only the emirates of Abu Dhabi and Dubai were included in calculations due to a lack of traveler data on the other 5 UAE emirates. Also, no MERS-CoV cases have been reported from these other emirates.

†OECD, Organization for Economic Cooperation and Development.

‡Estimates of population sizes were obtained from national statistics offices (*9*, *10*, *15–17*) while the number of travelers to each country was obtained from published data of the World Tourism Organization (Compendium of Tourism Statistics 2014 edition and Yearbook of Tourism Statistics 2014 edition) (*1*,*2*) and countries' visitor statistics (*3–5*). Data on the numbers of exported cases came from the World Health Organization MERS-CoV updates (*22*).

§The emerging economies of Chile, Mexico, and Turkey were excluded from OECD estimates. Estimates of the number of high-income OECD travelers for Qatar were based on total traveler estimates for Europe, North America, Australia and New Zealand.

¶For the analyses using travelers from all non-Middle Eastern countries, the average length of stay for travelers was estimated at 13.00 d for Saudi Arabia, 4.20 d for Jordan, 1.18 d for Qatar, and 3.65 d for Abu Dhabi and Dubai. For the OECD analyses, the average length of stay in Saudi Arabia was 10.71 d and 4.49 d for Abu Dhabi and Dubai. The Abu Dhabi and Dubai estimate was used for Qatar and Jordan for OECD analyses due to a lack of country-specific data and because Qatar, Jordan, and Abu Dhabi and Dubai had similar durations of stay for travelers from all non-Middle Eastern countries.

#Not all travelers' nationalities were listed individually in Abu Dhabi and Dubai data sources (*3*,*4*). Therefore, we produced conservative estimates of cases by including all travelers in the "Other Europe" and "Other South Asia" countries for Dubai. For Abu Dhabi, the top 25 countries of origin for travelers were listed in official statistics (*4*). Therefore, we conservatively assumed that the number of travelers from each of the unlisted countries was equal to the number of visitors from the 25th placed country.

**An additional case-patient was detected in the Republic of Korea (which was classified as a high-income country) but whose source country of infection was unclear (*23*). This case was included in the analyses of high-income countries and also of all non-Middle Eastern countries combined.

††Travel-associated cases that occurred among residents of the Middle East were included only in analyses comparing the rates of passive detection of importation events between high-income and other countries. Additional such cases were as follows: high-income countries – 1 case in Austria (*24*), 1 case in Greece (*25*), 2 cases in the United States (*24*,*26*); lower-income countries – 1 case in Turkey (*27*), and 2 cases in Thailand (*28*,*29*). A MERS-CoV case was also identified in a person who sought treatment in Germany for a respiratory infection (*30*). Our results for the difference in detection rates between high-income vs. lower-income countries were strengthened when this case was additionally included in analyses.

**Technical Appendix Table 2.** Sensitivity analysis for travelers' lengths of stay: estimated cumulative incidence of severe MERS-CoV cases across all Middle Eastern source countries during September 2012 – January 2016, using data for travelers from high-income OECD countries*

| Source countries | -2 Days LOS: Estimated cases (95% CI) | -1 Day LOS: Estimated cases (95% CI) | Average LOS†: Estimated cases (95% CI) | +1 Day LOS: Estimated cases (95% CI) | +2 Days LOS: Estimated cases (95% CI) |
|---|---|---|---|---|---|
| All countries | 5,463 (2,171–11,071) | 4,086 (1,623–8,280) | 3,263 (1,297–6,613) | 2,716 (1,079–5,504) | 2,326 (924–4,714) |
| Kingdom of Saudi Arabia | 3,799 (1,510–7,699) | 2,842 (1,129–5,758) | 2,269 (902–4,599) | 1,889 (751–3,828) | 1,618 (643–3,278) |
| Jordan | 809 (322–1,640) | 605 (240–1,126) | 483 (192–979) | 402 (160–815) | 345 (137–698) |
| Qatar | 273 (108–553) | 204 (81–414) | 163 (65–330) | 136 (54–275) | 116 (46–236) |
| Abu Dhabi and Dubai | 582 (231–1,179) | 435 (173–882) | 347 (138–704) | 289 (115–586) | 248 (98–502) |

*LOS, length of stay; OECD, Organization for Economic Cooperation and Development.

†The average length of stay of travelers from OECD countries in the 4 source countries was estimated to be 5.0 d.

**Technical Appendix Table 3.** Sensitivity analysis for travelers' lengths of stay: Estimated cumulative incidence of severe MERS-CoV cases in all Middle Eastern source countries (September 2012–January 2016) by using data for travelers from all non-Middle Eastern countries*

| Source countries | -2 Days LOS Estimated cases (95% CI) | -1 Day LOS Estimated cases (95% CI) | Average LOS† Estimated cases (95% CI) | +1 Day LOS Estimated cases (95% CI) | +2 Days LOS Estimated cases (95% CI) |
|---|---|---|---|---|---|
| All countries | 2,043 (1,061–3,499) | 1,683 (874–2,883) | 1,431 (743–2,452) | 1,245 (647–2,132) | 1,102 (572–1,887) |
| Kingdom of Saudi Arabia | 1,421 (738–2,434) | 1,171 (608–2,005) | 995 (517–1,705) | 866 (450–1,483) | 766 (398–1,312) |
| Jordan | 303 (157–518) | 249 (129–427) | 212 (110–363) | 184 (96–316) | 163 (85–279) |
| Qatar | 102 (53–175) | 84 (44–144) | 72 (37–123) | 62 (32–107) | 55 (29–94) |
| Abu Dhabi + Dubai | 218 (113–373) | 179 (93–307) | 152 (79–261) | 133 (69–227) | 117 (61–201) |

*LOS: length of stay; OECD: Organization for Economic Cooperation and Development

†The average length of stay of travelers from all non-Middle Eastern countries in the 4 source countries was estimated to be 6.7 d.
